# A Call to Action: Evidence for the Military Integration of Teledermoscopy in a Pandemic Era

**DOI:** 10.3390/dermatopathology9040039

**Published:** 2022-10-09

**Authors:** Gehan A. Pendlebury, John Roman, Vikas Shrivastava, Jerry Yuan

**Affiliations:** 1 College of Osteopathic Medicine, Nova Southeastern University, Fort Lauderdale, FL 33314, USA; 2 U.S. Naval Medical Center Portsmouth, Portsmouth, VA 23708, USA; 3 U.S. Naval Medical Center San Diego, San Diego, CA 92134, USA; 4 U.S. Naval Hospital Rota, 11530 Cádiz, Spain

**Keywords:** teledermatology, military medicine, operational dermatology, occupational skin disease, operational readiness, combat effectiveness, medical evacuation, mission preparedness, medical readiness, monkeypox pandemic, COVID-19 pandemic

## Abstract

Skin disease remains a common complaint among deployed service members. To mitigate the limited supply of dermatologists in the U.S. Military Health System, teledermatology has been harnessed as a specialist extender platform, allowing for online consultations in remote deployed settings. Operational teledermatology has played a critical role in reductions of medical evacuations with significant cost-savings. When direct in-person lesion visualization is unattainable, teledermoscopy can be harnessed as an effective diagnostic tool to distinguish suspicious skin lesions. Teledermoscopy has the versatile capacity for streamlined incorporation into the existing asynchronous telemedicine platforms utilized worldwide among deployed U.S. military healthcare providers. In terms of clinical utility, teledermoscopy offers a unique and timely opportunity to improve diagnostic accuracy, early detection rates, and prognostic courses for dermatological conditions. Such improvements will further reduce medical evacuations and separations, thereby improving mission readiness and combat effectiveness. As mission goals are safeguarded, associated operational budget costs are also preserved. This innovative, cost-effective technology merits integration into the U.S. Military Health System (MHS).

## 1. Background

Telemedicine harnesses technology-based software to deliver convenient and cost-effective medical care at a distance. Telehealth integration has been an ongoing evolution in the U.S. healthcare model. Telemedicine was introduced as a healthcare delivery model in the 1960s and has become a permanent delivery system [1]. Telemedicine consultations provide timely and cost-effective health care services in garrison and deployed settings. Telemedicine delivery can be broadly organized into four modalities: real-time (synchronous), store-and-forward (asynchronous), remote patient monitoring (RPM), and mobile health (mHealth) [2].

Real-time telemedicine consultations are conducted via synchronous video and synchronous audio. This modality supports direct visual communications between the provider and the patient. The asynchronous, or store-and-forward (S&F) method, involves the electronic delivery of medical information (i.e., documents, images, videos) [2]. RPM is a technology that allows healthcare providers to synchronously monitor their patients outside conventional clinical settings (at home or in a remote area). Physicians can remotely access patient data, including physical symptoms, chronic conditions, post-hospitalization rehabilitation, and laboratory results [2]. Lastly, mobile health (mHealth) is a type of telemedicine modality that utilizes smartphone applications for healthcare delivery. With the advancement of smartphones and other health-related electronic devices, this modality has gained significant momentum in medical care systems [2]. Mobile health applications support biometric data tracking: calorie intake, exercise duration, pulse, blood pressure, weight, calorie intake, and sleep patterns. Likewise, further data can be collected and promptly delivered to healthcare providers to help monitor their patients’ health.

For the last three decades, the United States Department of Defense (DoD) has recognized telemedicine as a component of the U.S. Military Health System (MHS) [3]. Military telemedicine aims to improve access to specialized health care services in areas where military treatment facilities are limited or unavailable [3,4]. Common modalities for military telehealth services include store-and-forward (asynchronous) systems, real-time (synchronous), and remote monitoring. Military dermatology frequently utilizes an asynchronous telemedicine model. Asynchronous delivery is a practical option for operational settings with a wide range of global time zones. Likewise, asynchronous delivery can be helpful to military dermatologists as photographs of cutaneous lesions can be further analyzed with computer software [4].

During the coronavirus disease-19 (COVID-19) pandemic, teledermatology gained unprecedented interest among healthcare providers worldwide [5]. Insurance companies continue to modify their policies on physician reimbursements for teledermatology services, which has supported the expansion of this technology. Strict pandemic quarantine measures have prevented patients in rural and underserved communities from receiving timely dermatological care [6]. Teledermatology has greatly increased access to dermatological care in the pandemic era [6].

Over the past three decades, the United States Military Health System has been utilizing teledermatology technology. Teledermatology has enabled more efficient consultations and assessments. Through telemedicine platforms, service members can see a specialist within an average waiting time of 12–24 h, in comparison to 4–8 weeks for in-person appointments [4]. Moreover, the DoD utilization of teledermatology has produced significant operational cost-savings. Military teledermatology has prevented unnecessary medical evacuations, facilitated urgent consults, and reduced physicians’ cost of travel [4].

Dermoscopy is a noninvasive visualization method that facilitates the early diagnosis of time-critical skin lesions (e.g., malignant melanoma and squamous cell carcinoma) by utilizing a dermatoscope [7,8]. A dermatoscope is a handheld device that contains an inbuilt illuminating system with a high magnifying power [7]. Similarly, teledermoscopy is a noninvasive diagnostic method that utilizes special instrumentation to assess cutaneous lesions when direct, clear visualization is challenging. Teledermoscopy is an invaluable option in the prevention, diagnosis, and treatment of dermatological conditions [7,8]. With technological advancements in digital photography, teledermoscopy can accurately refine diagnosis and improve disease prognosis. 

Teledermoscopy has the capacity for real-time remote visualization of skin lesions, thereby assisting in timely treatments [9]. Dermatologists can visualize skin lesions synchronously from different angles and observe changes with varying applications of pressure (blanching, non-blanching, pitting, or non-pitting) [10]. Several technological modalities enable teledermoscopy delivery, such as the combined utilization of a video camera (webcam or a smartphone) and a dermatoscope or magnifying glass. Other modalities include an “all-in-one” system dermatoscope with a built-in digital camera and a wireless digital microscope that can be connected to mobile phones and personal computers [10].

With an array of device options, teledermoscopy offers economical and diagnostic benefit in military health systems all over the world. However, to date, only one military research study has evaluated the clinical utility of teledermoscopy utilization in a military setting. This study was conducted in the United States (Navy Medical Corps) and evaluated the diagnostic efficacy of teledermoscopy in comparison to traditional in-person consultations with a board-certified dermatologist. This comparative study examined a variety of suspicious pigmented skin lesions and revealed matched diagnostic accuracy between remote teledermoscopic and traditional in-person evaluations. These preliminary findings offer invaluable clinical insight and allude to other benefits that have yet to be documented. Although teledermoscopy is a low-cost technology, there is scant research on its value in dermatological practice. Such limited literature underscores the need for further studies on teledermoscopy utilization, particularly in military healthcare settings [11]. 

We herein present a novel literature analysis to demonstrate evidence for the integration of teledermoscopy in military medicine. This specialized analysis provides an updated briefing on teledermoscopy research over the last two decades. In addition, this teledermoscopy report outlines: delivery options, diagnostic efficacy, training opportunities, calculable cost-saving benefits, clinical value in operational medicine, affordability of potential implementation, and overall relevance to mission-preparedness in a dynamic pandemic era.

## 2. Objectives

The main objective of this paper is to present organized evidence to illustrate the wide range of potential benefits associated with military utilization of teledermoscopy in a dynamically changing world with ongoing pandemics and other bioterrorism threats. This review is systematically organized to present data for the rationale of teledermoscopy application in support of mission effectiveness in the U.S. Military Health System. 

### Overview of Objectives

- Present multifaceted evidence for the integration of teledermoscopy to safeguard military missions and preserve operational budget costs.
- Report an updated briefing on teledermoscopy research over the last two decades.
- Outline modalities and other emerging technologies for teledermoscopy delivery.
- Highlight the clinical utility of teledermoscopy in early lesion identification and accurate diagnosis.
- Describe mission-related benefits associated with teledermoscopy utilization in operational settings: improved remote assessment of pigmented lesions and improved recognition when prompt biopsies are warranted.
- Highlight diagnostic longevity of teledermoscopy application: long-term management and surveillance of high-risk malignant skin cancers.
- Describe economic advantages associated with timely treatments in operational settings: reduced medical evacuations and separations.
- Depict incalculable benefits associated with accurate diagnosis and timely biopsies: improved prognosis, enhanced quality of life, and increased patient satisfaction.
- Underscore incalculable benefits due to avoided medical evacuations such as decreased dangers associated with traveling in potentially hostile environments.
- Outline potential mission strategies to overcome potential barriers in teledermoscopy military utilization.
- Highlight key roles of military dermatologists in operational settings: effective diagnostic strategies and timely treatments for potentially mission-disqualifying dermatological conditions.
- Discuss evolving trends that impact operational dermatology: mission readiness, combat effectiveness against pandemics, and other bioterrorism threats to security.

## 3. Materials and Methods

A literature search was conducted to extrapolate research articles on PubMed and MEDLINE on telemedicine and teledermatology. The literature search was designed to extract research articles with a specific focus on teledermoscopy. Therefore, “teledermoscopy” was the only key term utilized in the literature search. Publication dates were set from 2000 to 2022. Articles written in a language other than English were excluded. Abstracts, animal research, and pending papers were excluded from the literature search. The search retrieved a total of 95 articles. All articles were initially examined by a reviewer (GP) and were screened based on title and abstract. Articles were originally included if it contained the utilized key term in its title or abstract. Following the preliminary screening, 75 pertinent randomized controlled trials, systematic reviews, retrospective studies, prospective studies, cross-sectional studies, cohort studies, topic reviews, qualitative studies, comparative analyses, pilot studies, observational studies, books, and case series were included. No meta-analyses were identified (within the scope of the investigation) at the time of the literature search. 

## 4. Results 

Although a cost-effective technology, the comprehensive literature search dating over 22 years exposed scarce research on teledermoscopy and its clinical utility in dermatological practice. To date, only one research study has evaluated the diagnostic efficacy of teledermoscopy usage in a military setting. This milestone study was conducted in the United States (Navy Medical Corps) and revealed matched accuracy in remote teledermoscopic diagnosis compared to blinded, remote in-person evaluations with a board-certified dermatologist. Furthermore, this military study illustrated an inexpensive configuration option for the successful utilization of synchronous teledermoscopy in a military treatment facility. These promising results allude to opportunistic potential for operational dermatology. Such limited literature underscores the need for further research initiatives on teledermoscopy utilization in a military healthcare setting [11]. With an increasing array of device options, teledermoscopy offers economical and diagnostic utility in the United States military. 

## 5. Cost-Saving Benefits and Advantages of Military Teledermatology 

Teledermatology reduces medical costs associated with dermatological care in the military [4]. It is estimated that the healthcare costs associated with dermatology visits can be reduced by $35 million annually if approximately 5% of the office-based visits are shifted to teledermatology [12]. When compared to in-person evaluations, teledermatology offers matched diagnostic accuracy in skin disease [13]. The most common skin conditions requiring teledermatology consultation include acne, atopic dermatitis, hair loss, and skin cancer [14]. Of note, patients with suspicious lesions should always consult board-certified dermatologists for a full body skin examination. However, in-person dermatological evaluations may not be feasible for patients in deployed settings [15]. In such cases, general practitioners can photograph lesions of concern or conduct a synchronous teledermoscopy examination. Table 1 organizes evidence associated with teledermatology usage in military health systems. 

## 6. The Technological Evolution of Teledermoscopy 

The implementation of teledermoscopy can significantly improve early skin cancer detection than teledermatology alone [18]. Teledermoscopy can be harnessed as a practical diagnostic device to triage patients with skin conditions. With the advancement of technological delivery systems, primary care physicians and patients can conduct successful teledermoscopy sessions. Such advancements include smartphone-based microscopes, handheld digital microscopes, dermatoscopes with built-in camera systems, universal attachable dermatoscope lenses, and manual webcam attachments [18]. 

Smartphone microscopes are inexpensive and can provide high-quality diagnostic accuracy. These portable tools are widely available [21,22,23]. Smartphone microscopes are small magnifying lenses that attach to the back camera of a smartphone. Smartphone microscopes have a built-in illumination system and offer up to 40× magnification. These devices are travel-friendly, inexpensive, and can be efficiently utilized by general practitioners to transfer photos of suspected lesions [18,21]. This practical process can aid in the elimination of unnecessary in-person consultation (and associated costs). Likewise, smartphone microscopes can expedite referral for skin lesions that appear malignant. A study by Veronese et al. revealed an 80% diagnostic accuracy of a smartphone microscope device (NurugoTM) compared to a conventional dermatoscope [18]. These favorable results underscore the prudence for continued research on teledermoscopy. 

In addition to smartphone microscopes, handheld digital microscopes are harnessed as a viable modality. Likewise, hybrid dermatoscope devices offer teledermoscopy delivery via an “all-in-one” system without requiring a direct contact application with a webcam, smartphone, or another camera device. The costliest of all described technologies, “all-in-one” dermatoscopes offer the superior convenience of using one compact tool and enable smartphone connectivity via Bluetooth capabilities. Likewise, universal dermoscopy lenses are designed to magnetically attach to various image-capturing devices: virtually any digital single lens reflex camera, compact point-and-shoot systems, mirrorless system cameras, and smartphone devices. These portable lenses offer the unique convenience of multiple device compatibility, including smartphone devices. 

Additionally, a webcam in combination with a dermatoscope (or magnifying glass) can be utilized to visualize skin lesions during teledermatological consultations. Webcam devices can be readily employed to practice teledermoscopy. Likewise, all military dermatologists possess a dermatoscope and use dermoscopy in their daily practice [11]. The ubiquitous presence of webcam technology and personal dermatoscopes bypasses the need for high financial investments for initial military implementation [11]. The widespread existence of webcam technology at MTFs offers a unique learning opportunity for graduate medical education. Basic practice with a combined dermatoscope and webcam application has the potential to boost dermoscopy skills in military residency training programs. 

**Figure 1:**
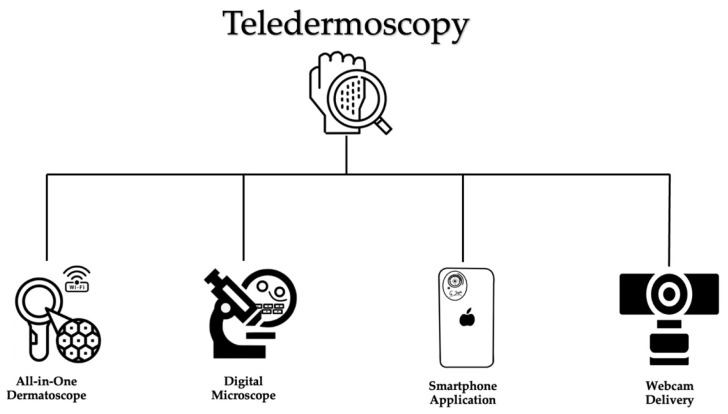
Teledermoscopy delivery. Overview of device options for teledermoscopy delivery.

Lastly, manual webcam attachment uniquely supports synchronous and asynchronous teledermoscopy delivery. An additional advantage, the manual attachment to webcams is not dependent on smartphone technologies. Furthermore, unlike smartphones, webcams can operate on minimal bandwidth (equivalent to one bar of data service). Such minimal bandwidth requirements support asynchronous delivery on the Defense Health Agency (DHA) Global Teleconsultational Portal (GTP) [11]. These simple yet effective features of webcam attachment delivery champion this modality as an affordable and pragmatic option for more widespread utilization of operational teledermoscopy in the United Military Health System [11]. Figure 1 provides an overview of different technologies employed in the delivery of teledermoscopy. Table 2 illustrates the distinct benefits and limitations of each teledermoscopic modality.

## 7. Teledermoscopy: Evidence for Military Implementation 

### 7.1. Synchronous Teledermoscopy Study in U.S. Military Training Facility 

The United States Department of Defense has utilized telemedicine for over three decades and has significantly improved dermatology care for deployed service members [3]. Moreover, teledermatology consultation in military treatment facilities has the highest number of consults than any other specialty in the military [4]. Additionally, military dermatologists continue to expand teledermatology in the military by implementing new strategies for teledermoscopy delivery. 

**Figure 2:**
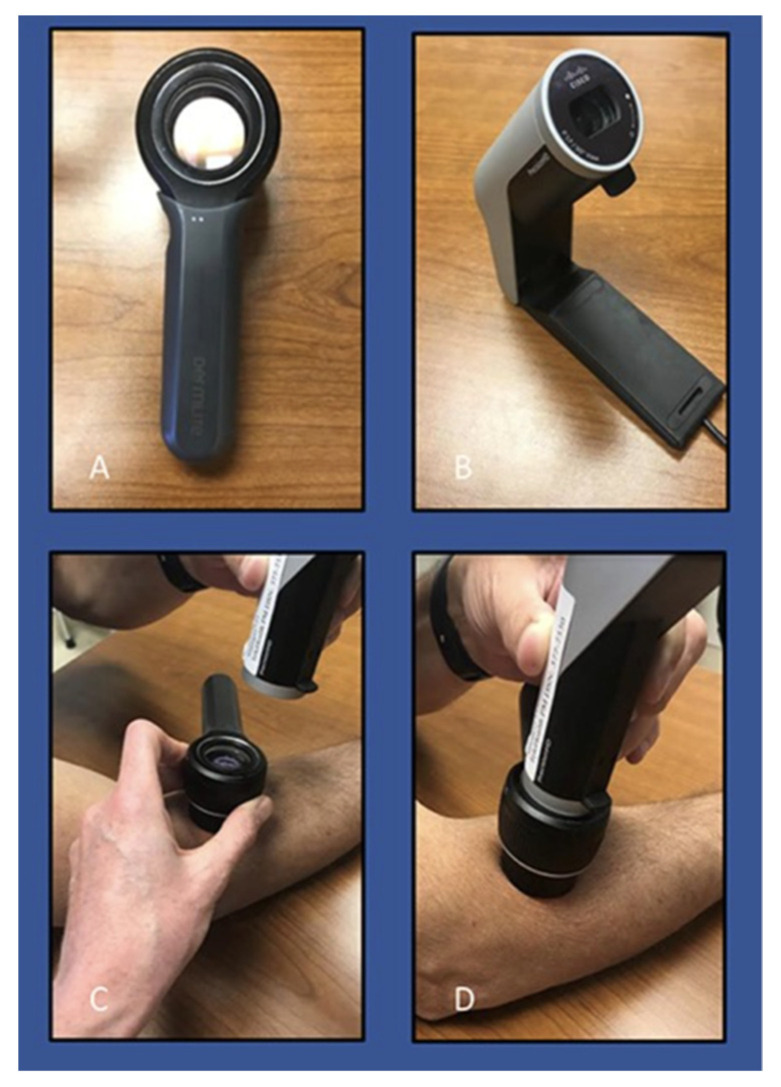
Synchronous teledermoscopy protocol tested at a military training facility. Basic configuration for teledermoscopy. (**A**) Dermatoscope; (**B**) Cisco Jabber Webcam; (**C**) Sanitize dermatoscope and skin lesions with alcohol; (**D**) Gentle contact between webcam and dermatoscope [11].

Day et al. conducted the first military study (Navy Medical Corps) to evaluate teledermoscopy within a military health system worldwide. The study sought to compare the diagnostic accuracy of a teledermatology consultation using synchronous teledermoscopy and a traditional in-person dermatological consultation [11]. The study was conducted on two patients at a remote military medical clinic, and each provider was blinded to the lesion diagnosis. Each patient presented with two lesions (benign and malignant) that were examined by two board-certified dermatologists [11]. The teledermoscopy session utilized a repurposed webcam ubiquitous at most military training facilities (MTFs) and a dermatoscope (×10 magnification) with transillumination properties. Figure 2 depicts the primary protocol used for the synchronous teledermoscopy application. This military research study revealed both the in-person dermatologist and the remote dermatologist were in 100% concordance regarding the accurate diagnosis of each lesion as benign or malignant when using teledermoscopy [11]. 

Current practice standards for military telemedicine in a deployed setting involve the utilization of an asynchronous “store & forward” teledermatology format. Given the spread of worldwide time zones, the S&F asynchronous format has mobilized specialized care in the military. Deployed providers typically email a raw picture with a brief description to a centralized email address which forwards to military dermatologists. 

While this study utilized a dermatoscope to visualize skin lesions, a basic magnifying glass (coupled with webcam) is a feasible option when dermatoscopes are otherwise unavailable. The versatility of teledermoscopy devices underscore its feasibility and affordability to general practitioners. This practical teledermoscopy delivery a sustainable option in operational settings with limited resources. Prompt visualization of dermatoscopic structures can benefit the timely diagnosis and treatments for deployed servicemembers. 

### 7.2. Histopathological Correlations 

**Figure 3:**
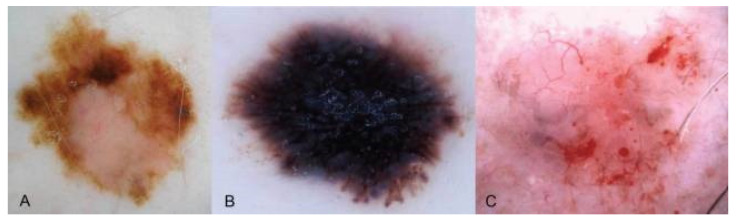
Dermoscopic images: (**A**) Dysplastic Nevus; (**B**) Melanoma in Situ; (**C**) Basal Cell Carcinoma (low grade-nodular type) [28].

Dermoscopic images provide greater clarity and detail for melanocytic lesions. Dermatoscopes contain valuable properties (e.g., epiluminescence microscopy), which can provide vital insight into dermoscopic structures [24]. Dermoscopic structures have histopathological correlations, a critical differentiating factor between benign versus malignant lesions and associated prognoses [24]. Earlier visualization of dermoscopic findings assists rapid lesion identification in military telemedicine [25]. Figure 3 demonstrates the visualization of benign and malignant lesions using teledermoscopy.

The IMAGE IT Trial analyzed approximately five hundred skin lesions of concern independently assessed through teledermoscopy and in-person consultations [24,25,26]. Out of 491 lesions of concern, only twelve malignant lesions were misreported as benign. However, when histopathology was utilized, only one malignant lesion was missed (one basal cell carcinoma misdiagnosed as a solar keratosis lesion) [25]. The IMAGE IT Trial determined teledermoscopy evaluation accuracy approximated 100% sensitivity and 90% specificity. The IMAGE IT Trial was conducted over a decade ago, and teledermoscopic technology has since exponentially advanced, suggesting even greater promise for excellent diagnostic reliability and successful implementation into clinical practice [25]. Likewise, teledermoscopy offers diagnostic longevity for the long-term management and surveillance of patients at high risk for cutaneous malignant melanoma [27]. 

Teledermoscopy application improves the remote assessment of pigmented lesions and synergistically determines the potential need for prompt biopsy [24,26]. Correct diagnosis and timely biopsies lead to incalculable benefits, including improved prognosis, quality of life, increased patient satisfaction, and decreased medical separation from military service.

### 7.3. Calculated Cost-Savings in Operational Settings 

Improved clinical outcomes ultimately reduce the need for medical evacuations, thereby improving combat effectiveness. Compared to the costs associated with dermatological evaluations overseas, the financial costs of evacuations overwhelmingly exceed in terms of fiscal impact [16,24,26]. A 2010 study evaluated a total of 2197 store-and-forward teledermatology consults generated by military health providers between the specified time frame: January 2005 through January 2009. Each dermatological consultation followed the same process: deployed healthcare providers emailed digital kodachromes with a brief clinical history to one centrally monitored email address. All emails were then triaged to the on-call consulting dermatologist [16]. 

This study calculated the costs associated with medical evacuation to be exponentially far greater than the costs related to providing specialized medical care. A summation of 1.4% (*n* = 40) of the dermatological teleconsultations recommended evacuation back to the United States for an estimated cost of $562,380 [16]. The total costs associated with medical evacuation included lost duty days, ground transportation, helicopter airlifts, extra personnel required security and transportation, meals, and temporary housing during evaluation and treatment [16]. The calculated analysis also revealed significant cost-savings associated with avoidance of medical evacuations [16]. With the utilization of teledermatology, 2157 military personnel were treated in Iraq, resulting in an estimated total cost-savings of 30.4 million dollars [16]. Teledermatology utilization also provides incalculable savings by avoidance of dangerous travel in a war zone [16]. These results underscore the magnitude of cost-savings associated with preventing medical evacuations in combat [16]. Additional mission benefits that cannot be fully quantified include cost-savings associated with enriched quality of life-related to earlier care access and disease management [29,30,31]. 

## 8. Teledermatology: Applications in a Pandemic Era

With unprecedented restrictions on societies, teledermatology has become increasingly utilized in a pandemic era [32]. With limited in-person interactions, teledermatology has become a vital alternative to in-person dermatology visits during global pandemics [33,34]. Teledermatology has made it easier for patients suffering from chronic skin conditions to continue seeing their dermatologists despite strict quarantine measures [34]. Additionally, teledermatology can be utilized to manage common ambulatory dermatoses and assess cutaneous manifestations of COVID-19 infections [33]. Such cutaneous manifestations include morbilliform rash, varicella-like rash, and livedo reticularis [14]. Many of these mild cutaneous conditions resolve spontaneously without medical intervention [14].

Additionally, with the recent emergence of the mutated Monkeypox virus, new cutaneous conditions continue to develop [14,35,36,37]. The rash associated with Monkeypox infection begins as a maculopapular rash that transforms into vesicles that scab and crust. The most common anatomical locations of the Monkeypox virus include the face, extremities, groin, and other body parts [35,36]. Currently, there is no standard treatment protocol for the Monkeypox virus. It is theorized that the Smallpox vaccine may protect against the Monkeypox virus. However, more research is needed to investigate the safety and confirm the efficacy of the proposed immunity. 

As of August 2022, forty cases of Monkeypox have been identified across the Department of Defense [36]. The Navy and Marine Corps Public Health Center (NMCPHC) designed a specialized reporting system to ensure situational awareness and DoD-wide collation and tracking of Monkeypox infections. According to NMCPHC officials, the risk of Monkeypox infection among military personnel remains low [35,36]. The need for military dermatologists will steadily rise as the global landscape continues to shift. Military dermatologists are critical in correctly diagnosing and treating pandemic-related cutaneous conditions and operational skin lesions. As the ongoing need for military dermatologists increases much faster than provider availability, teledermatology remains a precious resource in the military health system. 

## 9. Teledermoscopy: Opportunities to Address Healthcare Disparities 

Beyond operational settings, teledermoscopy has a fitting role in rural regions, as remote areas experience significant shortages of access to board-certified dermatologists [33]. Many patients in these rural regions already have limited access to primary care providers. Likewise, patients in these regions cannot readily consult dermatologists for necessary evaluations of chronic and acute skin conditions [33]. Due to such high demand and limited accessibility, obtaining an in-person dermatological consultation may take weeks to months. The mobilization of teledermatology offers a lifeline to rural areas.

Furthermore, teledermatology increases access to specialized care in underserved niches, including correctional facilities and migrant camps [38,39]. The societal impact of the COVID-19 pandemic underscores the unprecedented and continued need for teledermatology expansion, particularly in rural settings [34]. To support public health needs, dermatologists should incorporate teledermatology into their practice model to help address dermatological care disparities in remote and underserved areas.

## 10. Teledermoscopy: Strategies for Successful Military Integration

Teledermoscopy offers clinical utility in military medicine, with promising potential to support the medical readiness of the force. Despite feasible telehealth platforms for military implementation, operational settings present unique obstacles in telehealth delivery, including limited bandwidth, ongoing staff permanent change of station (PCS) assignments, staff inexperience with dermoscopy, and limitations in authorized reimbursable medical allowance to military providers. Dermatoscopes are not currently authorized as reimbursable equipment in the designated medical allowance for deployed medical providers. 

### 10.1. Solutions for Limited Bandwidth Connectivity 

Existing technologies can mitigate obstacles in limited bandwidth connectivity. In terms of technological implementation, teledermoscopy fits appropriately into the Defense Health Agency (DHA) Global Teleconsultational Portal (GTP) [40,41,42]. Enacted in 2021, GTP is an asynchronous virtual medical center (VMC) currently utilized in the United States Military Health System (MSH). GTP was derived from the merger of the Pacific Asynchronous TeleHealth and the Health Experts Online Portal (PATH/HELP) systems which previously enabled provider-to-provider teleconsultation and patient support to the MHS. Since 2004, GTP has operated as a worldwide accessible, HIPAA-compliant, secure, web-based system for military health care personnel and providers [42]. GTP has been widely used to access medical specialists, such as board-certified military dermatologists [42]. 

Interestingly, GTP can operate on a web browser with limited internet bandwidth, affording the unique opportunity to be utilized at military treatment levels [42]. Limited bandwidth due to poor data service can limit the transmissibility of medical data. As such, these technological adaptations allow for continued streaming at limited bandwidths via the Defense Health Agency Global Teleconsultational Portal, which operates on low bandwidth connectivity – equivalent to “one bar of service” in cellular usage [42]. 

### 10.2. Research-Based Training Methods for Improving Teledermoscopy Diagnosis

A comparative study assessed teledermoscopy versus clinical examination in the accurate identification of skin cancers. Results revealed high diagnostic accuracy of skin cancers via teledermoscopy. Additionally, this investigation demonstrated consistent diagnostic accuracy among general practitioners and surgeons [28]. Various factors attributed to proper diagnostic consensus including a six-hour training course, photography quality, systematic methodology, ABCDE clinical algorithm implementation, and data acquisition software [28]. 

The research study implemented a training course focused on two major domains: clinical diagnosis of skin cancer (per the ABCDE clinical algorithm) and proper acquisition of teledermoscopic images. The ABCDE algorithm taught providers important clinical factors in the assessment of suspicious lesions. A skin lesion was deemed suspicious if a minimum two of the following three criteria were graded positive: A — asymmetry; C — color; E — evolution. Training for proper acquisition of teledermoscopic images included hands-on training to utilize a teledermoscopic system: digital dermatoscope and specialized teledermoscopy data acquisition software. Providers were also taught to collect relevant clinical data points (patient identification, Fitzpatrick skin phototype, skin cancer risk factors, anatomical region of concern, relevant ABCDE criteria) to produce a teledermoscopic report. Figure 3 illustrates quality dermoscopic images obtained in the study [28]. These successful findings in diagnostic accuracy are suggestive to the spectrum of options to deliver focused teledermoscopy training for general providers. Selective teledermoscopy education could yield great benefits for military healthcare providers, especially those in critical settings where dermatologists are in limited supply. 

### 10.3. Solutions for Military Staff Relocation & Inexperience with Dermoscopy 

**Figure 4:**
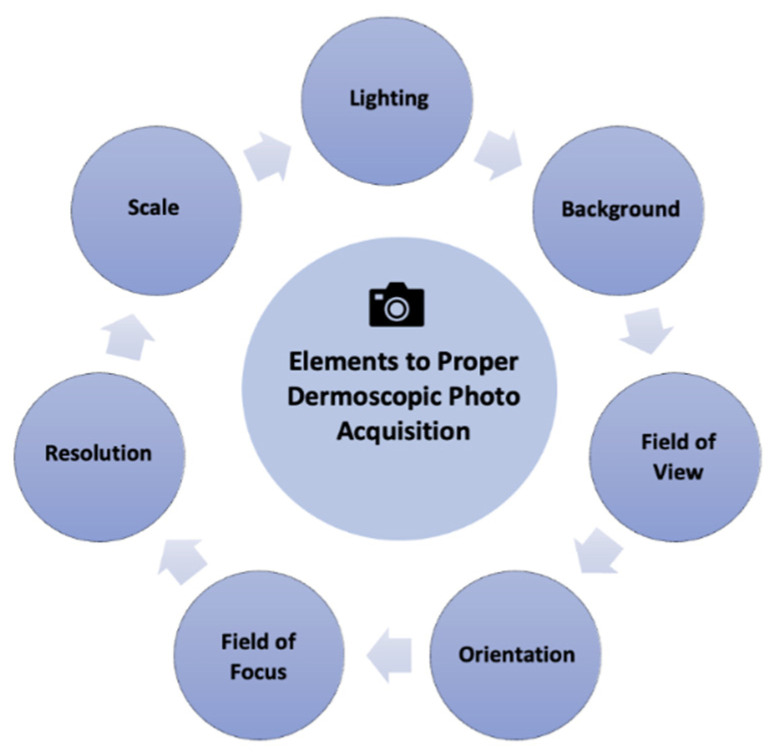
Key elements for the successful acquisition of clinical images for asynchronous teledermatology consultations. Important delivery considerations include appropriate lighting, background, field of view, orientation, focus, field, resolution, and photo scale.

The transient nature of military staff assignments leads to more frequent staff turnover. This recurrent turnover may lead to new medical support staff with little to no experience in teledermoscopy. Such inexperience may impede successful teledermoscopy delivery. Nonetheless, these barriers can be overcome by concise training to ensure basic competency. For example, a pragmatic option for teledermoscopy training could involve a demonstration video that teaches proper utilization for best diagnostic outcomes. Figure 4 features key points for ideal acquisition of dermoscopic images. 

**Figure 5:**
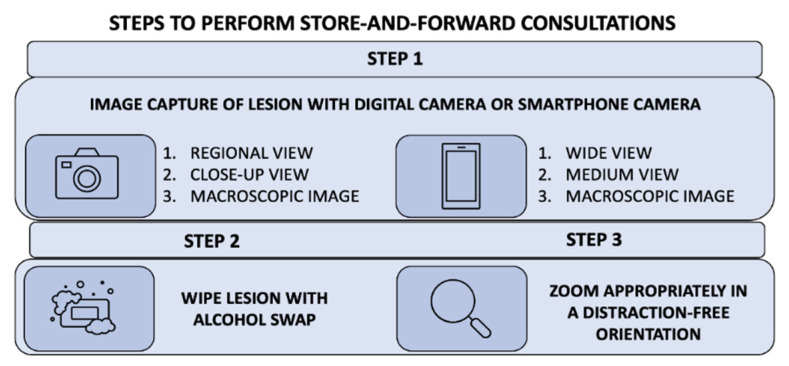
Teledermoscopy using an asynchronous delivery system. The summary of three key steps describes how to perform teledermoscopy via an asynchronous store-and-forward telemedicine consultation successfully. Asynchronous teledermatology is widely utilized in operational dermatology.

**Figure 6:**
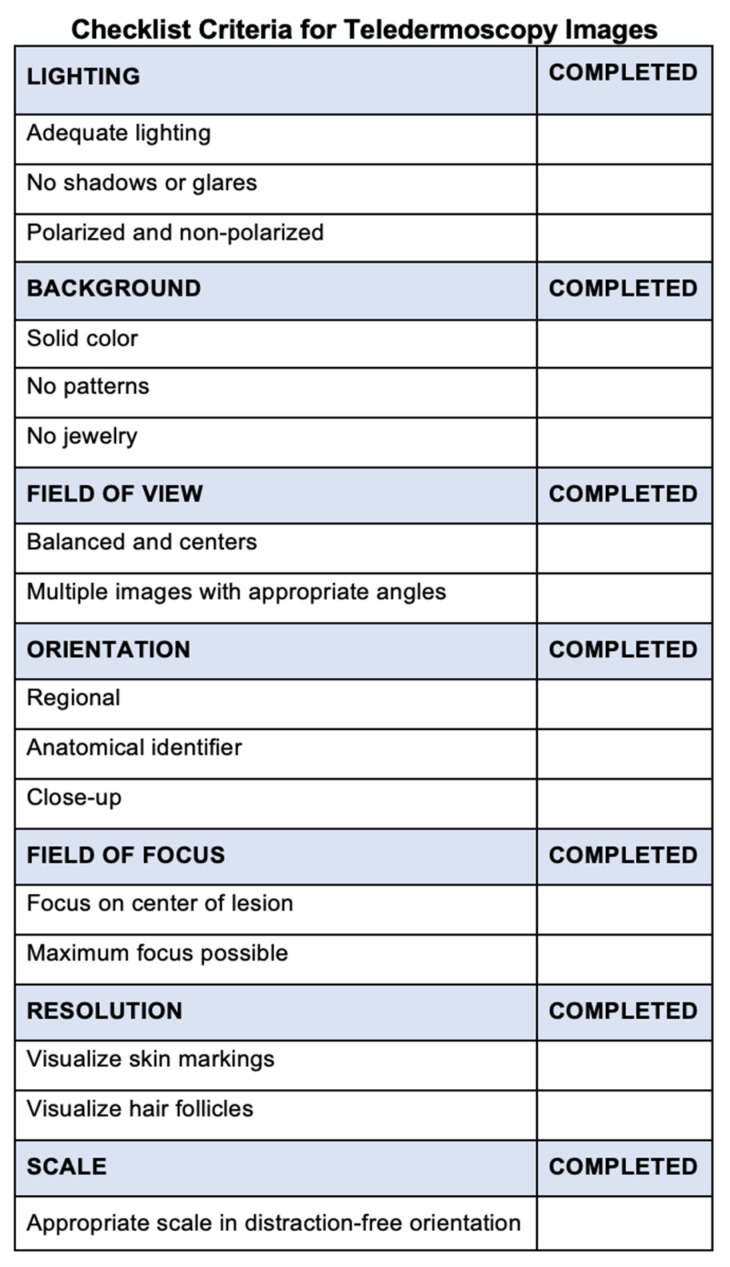
Checklist criteria for teledermoscopy images. The checklist provides recommendations to ensure high-quality teledermoscopic images via an asynchronous store-and-forward telemedicine consultation. Important delivery considerations include appropriate lighting, background, field of view, orientation, field of focus, resolution, and orientation.

Figure 5 outlines best practices to capture teledermoscopic images for asynchronous delivery, commonly used in operational dermatology. Figure 6 provides a checklist of recommendations to ensure high-quality teledermoscopic images via an asynchronous store-and-forward telemedicine consultation. Important delivery considerations include appropriate lighting, background, the field of view, orientation, focus, field, resolution, and photo scale. The implementation of dermoscopy algorithms can increase the sensitivity, specificity, and early detection of critical lesions, including skin cancers, compared to examination by the naked eye [43]. Likewise, dermoscopy can lead to the detection of thinner and smaller cancers. Such a diagnostic sensitivity is critical to mission effectiveness and decreased evacuations. With basic instruction, operational teledermoscopy could be successfully incorporated into the existing “store-and-forward” asynchronous military telemedicine platform.

### 10.4. Dermatoscope Inclusion on Authorized Medical Allowance List

A valuable strategy for successful military integration of teledermoscopy includes the necessary inclusion of a dermatoscope in the designated Authorized Medical Allowance List (AMAL) for deployed personnel. A traditional dermatoscope can be harnessed into a valuable tool for teledermoscopy. Usage of a traditional dermatoscope can be readily implemented into the existing “store and forward” asynchronous military telemedicine healthcare delivery system for deployed personnel. 

Military personnel often engage in operational requirements that increase their ultraviolet light exposure [44]. Likewise, sunscreen protection and sun-protective clothing are underutilized among military personnel, particularly in a deployed setting [45]. Furthermore, military (current and prior service) demographics include two groups who are predisposed to high rates of skin cancer: Caucasians (Fitzpatrick phototype II) and men over the age of 50 [46]. With the compounded risks of skin cancer among military personnel, it is prudent to include dermatoscopes as part of the designated medical allowance for deployed military providers. Dermatoscopes empower providers to detect lesions earlier in the disease course, significantly improving lesion prognosis and associated disease mortality reduction. With webcam technology readily available at most military training facilities (MTFs), teledermoscopy merits inclusion in the utilization of military telemedicine [11].

## 11. Conclusions

In military operational settings, primary healthcare providers provide most dermatologic care yet remain in limited supply. Due to the high demand and limited availability of dermatologists, teledermatology is an exceptional specialist extender that allows medical providers worldwide access to dermatology consultations. Teledermatology plays a critical role in reduced medical evacuations and associated cost-savings. As a valuable diagnostic feature of teledermatology, teledermoscopy integration into the military healthcare system is merited. With versatile diagnostic capacity, teledermoscopy can provide synchronous real-time remote visualization of skin lesions, facilitating faster diagnosis, timely treatments, and reduced medical evacuations. Likewise, teledermoscopy can be readily integrated into a “store-and-forward” asynchronous teledermatology platform, as commonly utilized in the United States Military Health System (MHS). 

As society continues to evolve in a pandemic era with bioterrorism threats, the United States Military Health System must also adapt to meet the critical need for swift diagnosis and prompt treatment. Currently, no teledermoscopy practice guidelines exist, presenting a unique leadership opportunity to establish clinical standards. Such initiatives could set a meaningful precedent for the nation to follow suit and improve prognosis of potentially fatal dermatological conditions. Improved clinical outcomes ultimately reduce medical evacuations, thereby improving medical readiness and combat effectiveness. As mission goals are safeguarded, associated operational budget costs are also preserved.

## Figures and Tables

**Table 1:** Evidence of Teledermatology Benefits in Military Medicine.

Benefits	Research Evidence
Healthcare Cost Reduction	- Minimize unnecessary medical evacuation [16] - Reduces dermatology referrals [17]
Timely Treatments	- Triage of more urgent, complicated medical problems [16] - Faster diagnostic procedures, medical treatment, and surgery [18]
Medical Care Mobilization	- U.S. VHA ^1^ implementation increased access to care ^2^ [19] - Face-to-face dermatological care is maintained virtually [20]
Early Lesion Detection	- Promotes timely treatment [16] - Improved prognosis [18] - Helpful triage for general practitioners [16,18]
Reduced Medical Evacuations	- Reduced time away from missions - Reduced appointment time to visit - Improved recovery time - Improved prognosis - Reduced medical discharge [16]

^1^ United States Veterans Health Administration. ^2^ From 2002–2014, the VA provided 34,928 teledermatology consultations (6% of all consultations) to veterans without local access to dermatologists.

**Table 2:** Teledermoscopy technologies. Summary of distinct benefits and limitations.

Teledermoscopy Device	Depiction ^1^	Benefits	Limitations
Hybrid “All-in-One” Dermatoscope	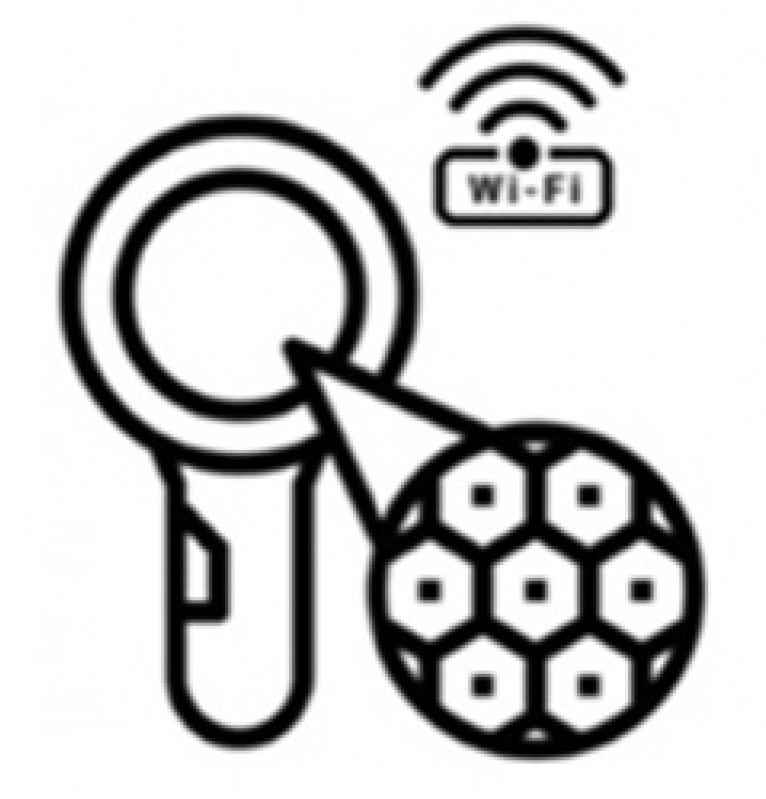	- Hybrid convenience - Smartphone connectivity - Robust diagnostic accuracy - Built-in illumination system - Up to 10× magnification - Travel-friendly - Polarization of image - Enables expedited referral	- Expensive ($1000 +) - Requires internet - Higher bandwidth - Less magnification - Not ideal for novice ^2^
Digital Microscope	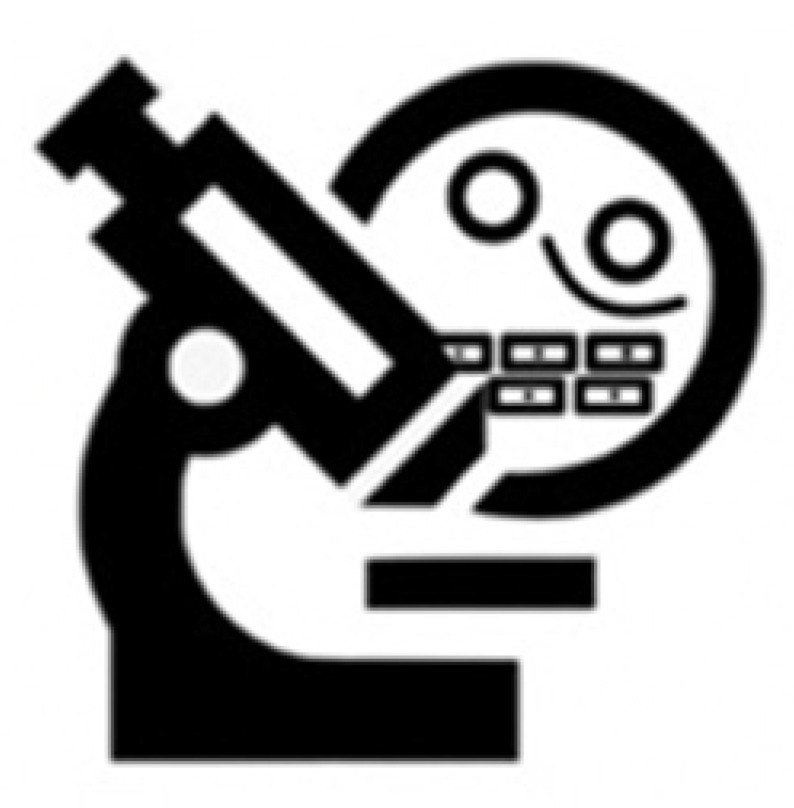	- Various utilization options ^3^ - Inexpensive (~$30) - Diagnostic accuracy - Wi-fi connectivity - 50×–1000× magnification - Photography and videography - High definition pixelation ^4^ - LED illuminance ^5^ - Supports expedited referral - USB-connectivity - Smartphone connectivity - Twistable knob to refine focus	- Wi-fi necessary - Higher bandwidth - Lacks polarization - Need reliable internet - Basic skills needed ^2^
Magnetic Attachment: Smartphone Application	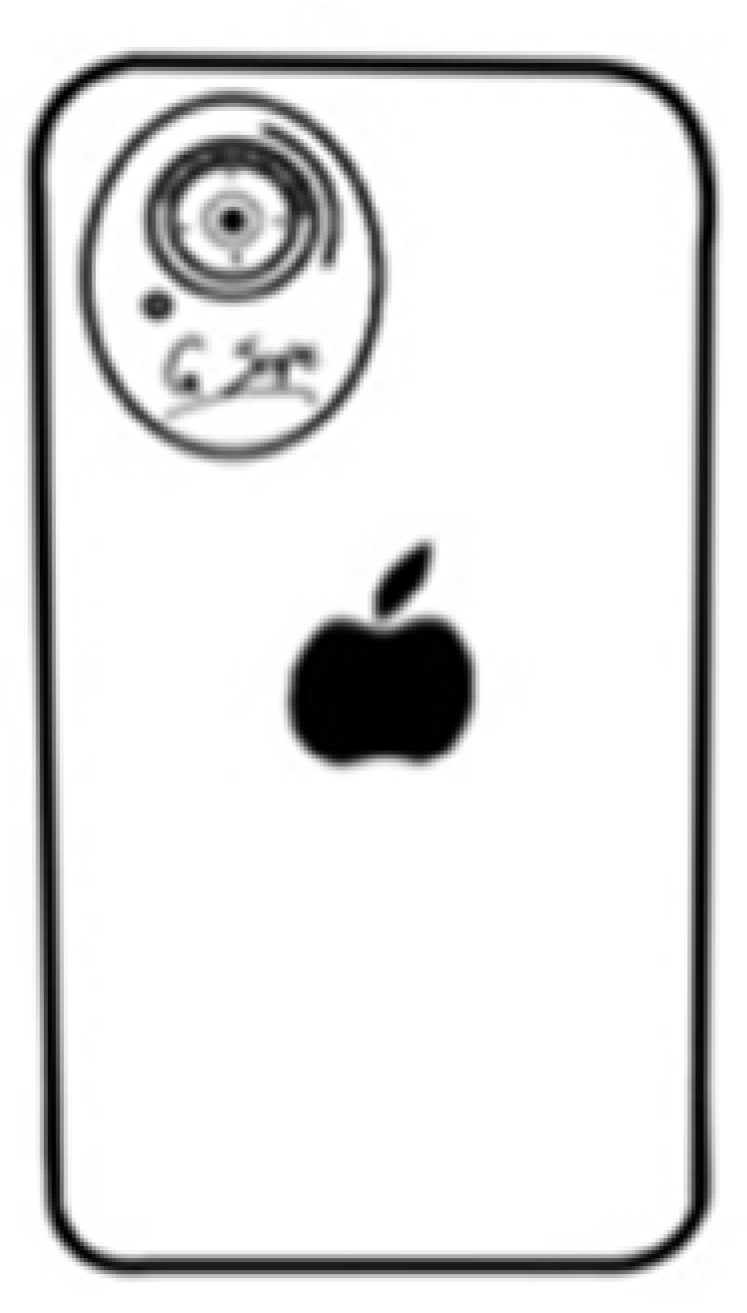	- Inexpensive (~$40) - Built-in illumination system - Polarization of image - Supports expedited referral - 50×−1000x magnification - Mobile application options - Simple electronic transfer - Attachable to dermatoscope - Does not require dermatoscope	- Requires internet - Higher bandwidth - Requires smartphone - Wi-fi dependence - Basic skills needed ^2^
Manual Attachment: Webcam Delivery	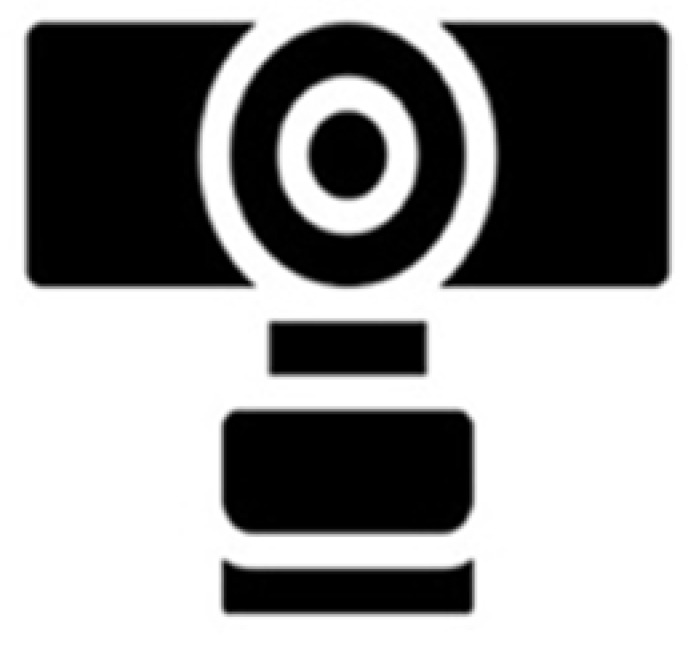	- Asynchronous/synchronous - Operates on limited bandwidth - Functional on limited data - Deployment-friendly - Present in many MTFs ^6^ - Inexpensive to implement ^6^ - Diagnostic accuracy - Built-in illumination system - Offers 400× magnification - Supports expedited referral - Polarization of image - Older webcams are usable - Operates without smartphone	- Dermatoscope usage - Limited access ^7^ - Financial costs ^8^ - Basic skills needed - Prior experience ^2^

^1^ Device depictions are for illustrative purposes only. ^2^ Prior dermoscopy experience or basic training in dermoscopy is recommended for proper usage. ^3^ Delivery options include microscope base or free mobile application with hand. ^4^ High pixelation includes 2 million pixels: high definition 1080P HD picture quality. ^5^ Light-emitting diodes (LED) contain properties of energy efficiency. ^6^ Basic webcam technology exists in military training facilities—cost-effective implementation. ^7^ In operational settings, dermatoscopes may not be readily available. ^8^ Dermatoscopes are currently not included in the reimbursable medical allowance for deployment.

## Data Availability

Not applicable.
